# Characterization of Black Lime (*Citrus* × *aurantiifolia*) Powder and Its Effects on the Physicochemical, Antioxidant, and Sensory Properties of Shortbread Cookies

**DOI:** 10.3390/molecules31183271

**Published:** 2026-09-15

**Authors:** Dariusz Dziki, Anna Krajewska, Waleed H. Hassoon, Agnieszka Nawrocka, Renata Nowak, Marta Olech

**Affiliations:** 1 Department of Thermal Technology, University of Life Sciences in Lublin, Głęboka 31, 20-612 Lublin, Poland; 2 Department of Food Engineering and Machines, University of Life Sciences in Lublin, Głęboka 28, 20-612 Lublin, Poland; 3 Department of Animal Production, College of Agriculture, Al-Qasim Green University, Babylon 51001, Iraq; waleedhassoon@agre.uoqasim.edu.iq; 4 Institute of Agrophysics, Polish Academy of Sciences, Doświadczalna 4, 20-290 Lublin, Poland; a.nawrocka@ipan.lublin.pl; 5 Department of Pharmaceutical Botany, Medical University of Lublin, Chodźki 1, 20-093 Lublin, Poland; renata.nowak@umlub.edu.pl (R.N.); marta.olech@umlub.edu.pl (M.O.)

**Keywords:** key lime, phenolic profile, flavonoids, FTIR spectroscopy, radical-scavenging activity, short biscuits, functional biscuits

## Abstract

Black lime (*Citrus × aurantiifolia*) is a dried key lime product traditionally used in Middle Eastern cuisine, characterized by an intense sour taste, distinctive citrus aroma, and dark color. Owing to its content of dietary fiber and bioactive compounds, particularly phenolics, black lime powder (BLP) may represent a promising ingredient for food enrichment. This study characterized BLP and evaluated the effects of replacing 3–15% of wheat flour with BLP on the physicochemical, antioxidant, spectroscopic, and sensory properties of shortbread cookies. Compared with wheat flour, BLP contained higher levels of dietary fiber, ash, and phenolic compounds, with protocatechuic, *p*-coumaric, vanillic, and ferulic acids, as well as isovitexin and vitexin, among the predominant compounds. Increasing BLP addition increased ash, soluble and insoluble dietary fiber, total phenolic content (TPC), and radical-scavenging activity. At 15% BLP, total dietary fiber reached 10.53% of cookie dry mass, while TPC and ABTS and DPPH radical-scavenging activities were approximately 5.5-, 11.1-, and 28.9-fold higher than in the control, respectively. These compositional enhancements were accompanied by technological changes, including increased moisture content and water activity, reduced cutting force, darker and less yellow cookies, and changes in starch organization and gluten structure indicated by FTIR analysis. Overall acceptability remained comparable with the control at 3–12% BLP but decreased at 15%, with 6–9% providing the most favorable balance between sensory quality and compositional enrichment. These findings indicate that BLP can be used as a fiber- and phenolic-rich ingredient for shortbread cookie enrichment.

## 1. Introduction

Increasing consumer interest in foods that provide benefits beyond basic nutrition has intensified research on reformulating widely consumed products with plant-derived ingredients. Ingredients rich in dietary fiber, phenolic compounds, vitamins, minerals, and other bioactive constituents are of particular interest because they can improve the nutritional value and bioactive compound content of foods [1,2]. Bakery products are suitable vehicles for such reformulation because they are widely consumed, readily available, and generally well accepted by consumers. Shortbread cookies are particularly attractive as a food matrix because of their high sensory acceptability, long shelf life, and simple formulation, which facilitates ingredient substitution. Conventional shortbread formulations contain high proportions of fat and refined sugar and relatively low amounts of dietary fiber and bioactive compounds; therefore, partial replacement of wheat flour with plant-derived materials may improve their nutritional and functional value [3,4]. However, enrichment can also alter dough behavior and cookie characteristics, including dimensions, color, texture, flavor, and consumer acceptability [3,4,5]. Previous studies have shown that unconventional flours, seeds, fruits and their by-products, vegetables, and herbal extracts can be incorporated into cookies to enhance their nutritional composition and antioxidant potential, although the optimal addition level depends on the technological and sensory effects of the ingredient [3,4,6,7,8,9].

Key lime (*Citrus* × *aurantiifolia*) is valued for its acidic taste and characteristic citrus aroma, as well as for its bioactive constituents [10]. The fruit and peel contain phenolic compounds, flavonoids, ascorbic acid, terpenoids, and limonoids, while organic acids—particularly citric acid—are largely responsible for the sour taste [10,11]. Lime essential oil is rich in volatile terpenes, with limonene, γ-terpinene, and β-pinene among its major constituents [12]. Together, these constituents contribute to the characteristic chemical and sensory profile of lime and support its potential use as an ingredient in food applications. Black lime, also known as loomi or lumi, is produced by drying whole ripe Key lime fruits, sometimes after blanching and/or brine pretreatment, until they become hard and dark [13]. Drying substantially reduces moisture content and modifies color, acidity, volatile composition, and overall flavor quality [13,14,15]. Importantly, black lime powder (BLP) differs from conventional citrus by-products such as isolated peel or pomace because it is produced from the whole fruit and therefore contains components originating from both the peel and internal tissues. Moreover, dehydration is not merely a moisture-removal step but can modify the chemical profile of lime. Previous studies have shown changes in the composition of volatile compounds during lime dehydration, including alterations in terpene fractions and reductions in several aldehydes, which may contribute to the characteristic aroma of the dried product [15]. Drying may also modify the extractability and stability of phenolic compounds, while the high content of structural polysaccharides in the whole-fruit material can influence water distribution and interactions with starch and proteins when incorporated into a cereal matrix. Thus, compared with conventional citrus by-products, BLP represents a chemically and structurally distinct ingredient whose effects in baked products may extend beyond simple fiber and phenolic enrichment. The resulting product has a pronounced sour taste, varying degrees of bitterness, and a distinctive dried-citrus aroma. In Middle Eastern cuisines, black lime is used whole or ground as a souring and flavoring spice; it is also combined with black tea and incorporated into regional spice mixtures such as baharat [13,14,16,17].

Previous research on the application of black lime in food products is scarce and has focused mainly on beverages. Altemimi et al. [18] investigated dried (black) Key lime juice processed using an electric-field pasteurization system, evaluating its physicochemical properties, phytochemical compounds, antioxidant activity, and microbial quality. Similarly, Aboud et al. [19] studied dried lime juice subjected to infrared processing, including its phenolic content, antioxidant activity, color, and sensory quality. Despite the widespread culinary use of lime, its application as a functional ingredient in bakery products has received relatively limited attention. Sasanka et al. [20] incorporated candied *Citrus aurantifolia* peel, together with banana flour, into biscuits, demonstrating the feasibility of using lime-derived material in a baked product. However, this approach involved candied peel rather than dried whole-fruit black lime powder and did not investigate its use as a partial wheat flour substitute in a conventional shortbread matrix. Thus, the effects of BLP on the physicochemical, structural, antioxidant, and sensory properties of shortbread cookies remain largely unexplored.

From a technological and nutritional perspective, BLP may be a valuable ingredient in shortbread cookie formulations because of its potential contribution to dietary fiber and phenolic compound content, pronounced acidity, dark color, and distinctive aroma. At the same time, these characteristics may produce substantial changes in the cookie matrix and affect texture and sensory acceptability. To the best of our knowledge, the use of BLP as a partial substitute for wheat flour in shortbread cookies has not previously been investigated. Based on the composition of black lime powder, particularly its dietary fiber, organic acids, and phenolic compounds, it was hypothesized that its incorporation into shortbread cookies would modify the physicochemical and structural properties of the product matrix, while increasing antioxidant potential and influencing sensory quality. Therefore, this study had two objectives: (i) to characterize BLP in terms of its proximate composition, dietary fiber content, color parameters, total phenolic content, antioxidant activity, and phenolic profile; and (ii) to determine the effects of partially replacing wheat flour with BLP on the physicochemical, spectroscopic, textural, antioxidant, and sensory properties of shortbread cookies.

## 2. Results and Discussion

### 2.1. Characterization of BLP and Wheat Flour

Table 1 compares the proximate composition, total phenolic content (TPC), antioxidant activity, and color of BLP and wheat flour. The two raw materials differed significantly in every measured parameter (*p* < 0.05). Wheat flour contained almost twice as much protein as BLP (9.93% vs. 5.15%), whereas BLP contained more fat (2.39% vs. 1.44%) and substantially more ash (5.10% vs. 0.48%). Available carbohydrates accounted for 33.62% of BLP and 85.97% of wheat flour.

The largest compositional difference concerned dietary fiber. BLP contained 53.75% total dietary fiber, approximately 25 times the concentration measured in wheat flour (2.17%). Both the insoluble fraction (29.78% vs. 1.19%) and the soluble fraction (23.97% vs. 0.99%) were markedly higher in BLP (Table 1). Notably, soluble fiber accounted for approximately 45% of the total fiber in BLP, indicating that BLP is not only a concentrated fiber source but also contains substantial amounts of both fiber fractions. From a nutritional perspective, these fractions may provide complementary physiological effects: insoluble fiber is generally associated with increased fecal bulk and intestinal transit, whereas soluble fiber may influence viscosity, fermentability, and interactions with the gut microbiota. The high fiber content may therefore increase the nutritional value of products enriched with BLP. However, it may also affect their technological properties, including water absorption, dough consistency, product volume, and texture, which should be considered when determining an appropriate substitution level.

BLP also contained approximately 26 times more total phenolic compounds than wheat flour. This difference was accompanied by approximately 39- and 77-fold higher antioxidant activity in the ABTS and DPPH assays, respectively, suggesting that phenolic compounds contributed substantially to the antioxidant potential of BLP (Table 1). The different magnitude of the responses obtained with the two assays may reflect differences in their reaction mechanisms and sensitivity to individual antioxidant compounds present in the samples. Nevertheless, these results demonstrate the potential of BLP as an ingredient that could simultaneously enhance the fiber content and in vitro antioxidant capacity of cereal-based products.

The raw materials also differed significantly in all color coordinates. The markedly lower lightness (L*) value of BLP confirmed its substantially darker appearance compared with wheat flour. Its higher redness (a*) indicated a stronger red component, whereas the higher yellowness (b*) value of wheat flour reflected its more pronounced yellow coloration. The darker and redder appearance of BLP may be associated with the presence of naturally occurring pigments and phenolic compounds. Among the pigments reported in lime, β-carotene and lutein are the predominant carotenoids [21], while phenolic compounds identified in *Citrus aurantiifolia* include flavonoids such as naringin, naringenin, hesperidin, and hesperetin [22]. In addition, the drying process itself may substantially affect lime color. Izli et al. [23] demonstrated that drying decreased the L* value of lime, resulting in a darker appearance, and shifted the a coordinate from negative values in fresh lime to positive values in dried samples, indicating a transition from greenness toward redness.

Overall, BLP was distinguished from wheat flour primarily by its high dietary-fiber, ash, and phenolic contents, its strong in vitro antioxidant activity, and its characteristic dark color. This composition indicates that BLP could serve as a multifunctional ingredient for improving the nutritional and antioxidant properties of cereal-based products.

### 2.2. Phenolic Profile of BLP

BLP exhibited a diverse profile of phenolic compounds, comprising phenolic acids, flavonoid aglycones, and flavonoid glycosides (Table 2). Phenolic acids predominated among the quantified compounds. Protocatechuic acid had the highest content (292.841 µg/g DW), followed by *p*-coumaric (171.541 µg/g DW), vanillic (166.632 µg/g DW), and ferulic (127.563 µg/g DW) acids. The remaining phenolic acids were present at lower levels, with gallic acid having the lowest content. Ali et al. [24] also reported the presence of protocatechuic, *p*-coumaric, ferulic, syringic, sinapic, and gallic acids in black lime. However, different compounds predominated among the acids reported in both studies: protocatechuic acid was predominant in the present study, whereas syringic and ferulic acids were the most abundant shared acids in the study by Ali et al. [24]. Among all phenolic acids quantified by those authors, ellagic acid, which was not determined in the present study, had the highest content. A direct comparison of the results is limited by methodological differences, particularly in the extraction procedures used. Ali et al. [24] employed a single-step extraction with acidified 80% methanol, whereas the present study used a two-step extraction procedure. These differences may have affected the extraction efficiency and, consequently, the concentrations of individual phenolic compounds detected. The results may also have been influenced by the origin and maturity of the fruit, as well as by processing and storage conditions.

The phenolic acid profile of black lime also showed partial agreement with the results reported by Czech et al. [25] for fresh lime fruit. The authors found that, among the phenolic acids analysed, chlorogenic, *p*-coumaric, and ferulic acids had the highest contents in whole fruit. Moreover, the contents of these acids were higher in the peel than in the pulp, indicating that the presence of the peel may substantially shape the phenolic profile of the whole fruit. Ding et al. [26] found that protocatechuic acid was one of the predominant non-extractable phenolic compounds in lemon slices and that drying affected the contents of both extractable and non-extractable phenolic fractions. Thermal processing may result in the degradation of thermolabile compounds while also increasing the contents of certain phenolic acids determined in extracts, possibly due to their release from bound forms and the consequent increase in their extractability [27,28]. The transformations occurring during black lime production may therefore have partially shaped the observed phenolic acid profile.

Flavonoid aglycones were present in black lime powder at substantially lower levels than phenolic acids, with isorhamnetin (9.574 µg/g DW) and apigenin (1.850 µg/g DW) being the predominant compounds in this group. Among the flavonoid glycosides, the sum of isovitexin and vitexin had by far the highest content (161.298 µg/g DW). Ali et al. [24] also reported the presence of isovitexin in black lime, whereas Phucharoenrak et al. [29] tentatively identified this compound in an ethanolic extract of *Citrus aurantifolia* peel. Both vitexin and isovitexin were also identified in a hydroalcoholic extract of the leaves of this species using HPLC-PDA-ESI/MS/MS, and their structures were confirmed by NMR [30]. However, owing to differences between plant tissues, these findings cannot be directly extrapolated to the fruit. Vitexin and isovitexin are isomeric C-glucosides of apigenin, in which glucose is attached to the C-8 and C-6 atoms of the apigenin skeleton, respectively [31]. The direct C–C bond between the glucosyl residue and the flavonoid skeleton is more resistant to acid and enzymatic hydrolysis than the C–O bond occurring in O-glycosides [31,32]. The greater resistance of the C-glycosidic bond to hydrolysis may have partially favoured the retention of vitexin and isovitexin during black lime production. Nevertheless, flavone C-glycosides are not completely resistant to high temperatures and may undergo degradation during intensive thermal processing [33]. Narcissoside (20.625 µg/g DW), luteoloside (6.759 µg/g DW), eriocitrin (4.441 µg/g DW), and narirutin (3.870 µg/g DW) were also quantified in BLP, although at lower levels.

The two-step extraction procedure enabled a more comprehensive characterisation of the phenolic compounds analysed in black lime. Most of the quantified compounds were extracted predominantly during the first step using 80% methanol, whereas the contribution of the extract obtained in the second step to their total contents was small. Nevertheless, the distribution of analytes between the sequentially obtained fractions was compound-dependent. Eriocitrin was extracted mainly during the second step, whereas narirutin was quantified only in the second fraction and gallic acid was detected exclusively in that fraction. The benefit of repeated extraction for improving narirutin recovery was also demonstrated by Kim and Lim [34], although they investigated a different citrus material and employed different extraction conditions. The first and second extraction steps with 58.4% ethanol recovered 82.4 and 14.8% of this compound, respectively, resulting in a total recovery of 97.2%. The present findings therefore indicate that, although the contribution of the extract obtained in the second step to the total content of the quantified compounds was small, this step reduced the risk of omitting certain analytes from the resulting profile.

### 2.3. Physical and Technological Properties of Cookies

BLP incorporation caused small but significant changes in cookie moisture content and water activity (Table 3). The control contained 3.80% moisture, significantly less than every BLP-enriched sample (4.07–4.21%). Relative to the control, BLP increased moisture content by approximately 7–11%. This response is consistent with the high dietary-fiber content of BLP and the capacity of citrus-derived fibers and pectins to retain water. A small increase in moisture was also observed when wheat flour was partially replaced with flour produced from bergamot by-products [35]. Drying conditions and particle characteristics can substantially affect the hydration properties of lime residues [36].

Water activity increased from 0.293 in the control to 0.415 after 3% replacement and then rose more gradually to 0.433 at 15% BLP. Hydrophilic fiber fractions may have altered water retention and distribution in the cookie matrix [30]. Nevertheless, all values were below 0.60, a range that does not support the growth of most bacteria, yeasts, and molds [37]. This observation concerns microbial growth potential at the measured water activity and does not replace a complete shelf-life or microbiological assessment.

Cutting force decreased progressively from 52.91 N in the control to 32.77 N at 15% BLP, a reduction of approximately 38%. The 3BLP and 6BLP samples did not differ significantly, and 9BLP overlapped statistically with both 6BLP and 12BLP. The concurrent rise in moisture and water activity may have contributed to softening of cookies because water plasticizes amorphous food components, increases molecular mobility, and can reduce crispness and fracture resistance [38]. Partial replacement of wheat flour also reduced the proportions of flour-derived starch and proteins involved in structure formation. Although gluten development is limited in fat-rich, low-moisture shortbread dough, flour components still contribute to the continuous baked matrix. Their replacement with hydrated fiber and plant particles may therefore have produced a more heterogeneous and less fracture-resistant structure. Pectin and fruit-skin powders can modify dough rheology and the distribution of water among starch, proteins, sugars, and fiber [39]. In addition, organic acids naturally present in lime, particularly citric acid [11], may modify the physicochemical environment of the dough. Citric acid has been shown to affect water absorption, solubility, and the mechanical properties of wheat-starch gels [40], while changes in citrate-buffer pH can influence gluten protein aggregation and secondary structure in wheat-flour dough [41]. Soluble pectic polysaccharides and other hydrophilic dietary-fiber components may additionally compete with starch and proteins for available water and participate in intermolecular interactions within the dough matrix [42]. These mechanisms are consistent with the FTIR observations obtained in the present study. In the polysaccharide region, the increased intensity of the band at 995 cm^−1^ up to 12% BLP suggested changes toward a more ordered starch structure [43], while shifts in bands associated with C–O, C–C, C–O–H, and glycosidic C–O–C vibrations indicated modifications of the starch environment and interactions between wheat starch and BLP-derived polysaccharides [44]. These observations are consistent with competition for water between starch and the hydrophilic fiber fraction of BLP and with altered local water distribution during baking. At the same time, changes observed in the amide III and amide I regions indicated substantial modification of the gluten secondary structure. The shift in the β-sheet region and the appearance of spectral features associated with random coils and protein aggregates suggest hydrogen-bond-mediated interactions between gluten polypeptides and BLP-derived polysaccharides, accompanied by partial reorganization of the native gluten secondary structure [45]. Such structural rearrangements, together with moisture plasticization, dilution of flour-derived structural components, and incorporation of hydrated fiber and pectic material, may contribute to the formation of a more heterogeneous and less mechanically resistant baked matrix, thereby providing a structural basis for the progressive decrease in cutting force observed with increasing BLP incorporation. However, the present measurements do not allow the individual contributions of these mechanisms to be quantified. The cutting-force results are consequently consistent with several interacting mechanisms, but the present measurements do not identify their individual contributions.

Cutting force has also been used as an instrumental indicator of cookie hardness [46]. In brittle products such as biscuits, mechanical resistance to fracture has been shown to be closely related to sensory hardness [47]. Although sensory crispness cannot be inferred directly from maximum cutting force alone, mechanical fracture characteristics have been shown to contribute to the perception of crispness in brittle foods [48]. Therefore, although the cutting-force measurement used in the present study does not provide a complete characterization of individual texture attributes such as fracturability or crispness, it provides a relevant comparative measure of the mechanical texture and hardness of the cookies.

BLP incorporation also affected selected technological properties of the cookies (Table 3). Spread ratio ranged from 5.32 to 5.63. Although the highest value was observed for 15BLP, it did not differ significantly from the control; significant differences were observed only between 15BLP and the 3BLP and 6BLP samples. Thus, BLP addition did not produce a clear concentration-dependent change in cookie spread relative to the control. Volume expansion showed a similar pattern. Values ranged from 62.73% to 72.46%, and no BLP-enriched sample differed significantly from the control. However, 15BLP showed significantly lower volume expansion than 3BLP.

Baking loss decreased slightly with increasing BLP incorporation, from 12.46% in the control to approximately 12.10–12.11% at 12–15% BLP. Cookies containing 9–15% BLP had significantly lower baking loss than the control, whereas 3BLP and 6BLP showed intermediate values. This reduction may be associated with the high content of hydrophilic dietary-fiber components in BLP, which can bind water and thereby limit mass loss during baking. This interpretation is also consistent with the higher final moisture content observed in BLP-enriched cookies.

### 2.4. Effect of BLP on the Proximate Composition of Cookies

BLP significantly altered cookie composition (Table 4). Increasing replacement was accompanied by gradual decreases in protein, and available carbohydrate contents. Protein declined from 7.66% in the control to 7.22% in 15BLP, consistent with the lower protein content of BLP than wheat flour. Available carbohydrates decreased from 58.9% to 50.47%. Fat content remained relatively stable across the formulations, ranging from 29.77% to 30.42% DW, and no significant differences were observed among the control and BLP-enriched cookies (*p* > 0.05). This is consistent with the relatively small difference in fat content between BLP and wheat flour and indicates that partial flour replacement with BLP had no meaningful effect on the lipid content of the final product. In contrast, ash and dietary fiber contents increased progressively with increasing BLP incorporation. Ash rose from 0.72% to 1.36%, soluble fiber from 0.62% to 4.18%, and insoluble fiber from 2.04% to 6.35%. Total dietary fiber therefore increased almost fourfold, from 2.67% in the control to 10.53% in 15BLP. These changes are consistent with the much higher ash and fiber contents of BLP than wheat flour and with reports that fiber-rich food-industry by-products increase the fiber and mineral fractions of wheat-based bakery products [49]. Thus, the clearest compositional effect of BLP was substantial dietary-fiber enrichment.

### 2.5. Effect of BLP on Cookie Color

BLP substantially changed cookie color, principally by reducing lightness and yellowness (b*) (Table 5). L* decreased from 73.22 in the control to 42.69 in 15BLP, demonstrating progressive darkening with increasing replacement level. This effect can primarily be attributed to the replacement of light-colored wheat flour with the inherently much darker BLP, as indicated by the color parameters of the raw materials. Similar reductions in cookie lightness following the incorporation of citrus peel and other plant-derived powders have previously been reported [50,51].

Non-enzymatic browning during baking, including Maillard reactions and sugar caramelization, may have additionally contributed to the final color; however, because browning products were not directly determined, this mechanism should be considered contributory rather than confirmed. The control cookies also exhibited the highest yellowness. Although the response was not strictly linear among the enriched formulations, BLP consistently reduced yellowness, probably because its brown–black constituents increasingly masked the yellow tone of the control cookie matrix. The non-linear variation in yellowness may reflect the combined effects of the intrinsic color of BLP, its distribution within the dough, and browning reactions occurring at the cookie surface. In contrast, redness did not differ significantly among the formulations, suggesting that BLP affected primarily lightness and yellowness rather than shifting the red–green color balance. Total color difference (ΔE) increased from 20.97 at 3% BLP to 34.61 at 15% BLP. Thus, even the lowest substitution level produced a readily perceptible difference from the control, while the progressive increase in ΔE confirms a strong, concentration-dependent visual impact of BLP incorporation. Such pronounced darkening may contribute to the distinctive appearance of black-lime cookies, although its effect on consumer acceptance should be interpreted together with the sensory color and overall-acceptability scores.

### 2.6. Effect of BLP on Total Phenolic Content and Antioxidant Activity of Cookies

BLP-enriched cookies had significantly higher TPC and antioxidant activity than the control in both radical-scavenging assays (Table 6). The response was dose dependent. At 15% replacement, TPC, ABTS activity, and DPPH activity were approximately 5.5-, 11.1-, and 28.9-fold higher, respectively, than in the control. TPC correlated strongly with ABTS (r = 0.9934; *p* < 0.001) and DPPH (r = 0.9905; *p* < 0.001), and the two antioxidant assays were also strongly correlated (r = 0.9922; *p* < 0.001). These associations, together with the high TPC and radical-scavenging activity of the raw material (Table 1), indicate that BLP was the principal source of extractable antioxidant constituents in the enriched formulations. The markedly higher fold increase observed for DPPH than for ABTS should not be interpreted simply as greater antioxidant activity measured by the DPPH assay. This difference was partly influenced by the very low DPPH value of the control sample (0.13 µmol TE/g DW) compared with the corresponding ABTS value (0.30 µmol TE/g DW). Consequently, at 15% BLP incorporation, the relative increases were 28.9-fold and 11.1-fold for DPPH and ABTS, respectively, whereas the absolute increases were much more similar (3.63 and 3.03 µmol TE/g DW, respectively). Moreover, DPPH and ABTS assays employ chemically different radicals and differ in steric accessibility, reaction kinetics, and sensitivity to solvent and reaction conditions; therefore, individual phenolic compounds and other reducing constituents of the cookie extract may contribute differently to the response of each assay [52]. The complex cookie matrix, containing phenolic compounds, lipids, proteins, carbohydrates, and thermally generated reaction products, may further influence the accessibility and reactivity of antioxidants toward the two radicals. Thus, the difference in fold response most likely reflects both the lower baseline DPPH activity of the control and assay-specific radical-scavenging chemistry rather than a proportional difference in the overall antioxidant capacity of the cookies.

Baking may have modified the measured response in addition to the direct contribution of BLP. Mixing and thermal treatment can alter phenolic extractability, including by releasing phenolic acids from bound forms [53]. Maillard-reaction products formed during baking can also show antioxidant activity in chemical assays [54]. However, thermal reactions may simultaneously generate compounds of concern, including acrylamide and 5-hydroxymethylfurfural. Consequently, higher in vitro antioxidant activity should not be interpreted as direct evidence of a health benefit. The present trend agrees with studies in which mosambi-peel extract [55], grapefruit-pomace powder [56], or orange-peel powder [57] increased TPC and antioxidant activity in wheat-based cookies. With 15% orange-peel powder, Al-Saab and Gadallah reported 7.2- and 9.5-fold increases in TPC and DPPH activity, respectively [57]. The corresponding increases with 15% BLP were 5.5- and 28.9-fold. However, direct quantitative comparisons between studies should be interpreted cautiously due to differences in raw material composition, cookie formulation, extraction conditions, baking parameters, and analytical methods.

### 2.7. FTIR Analysis of Cookies

**Figure 1:**
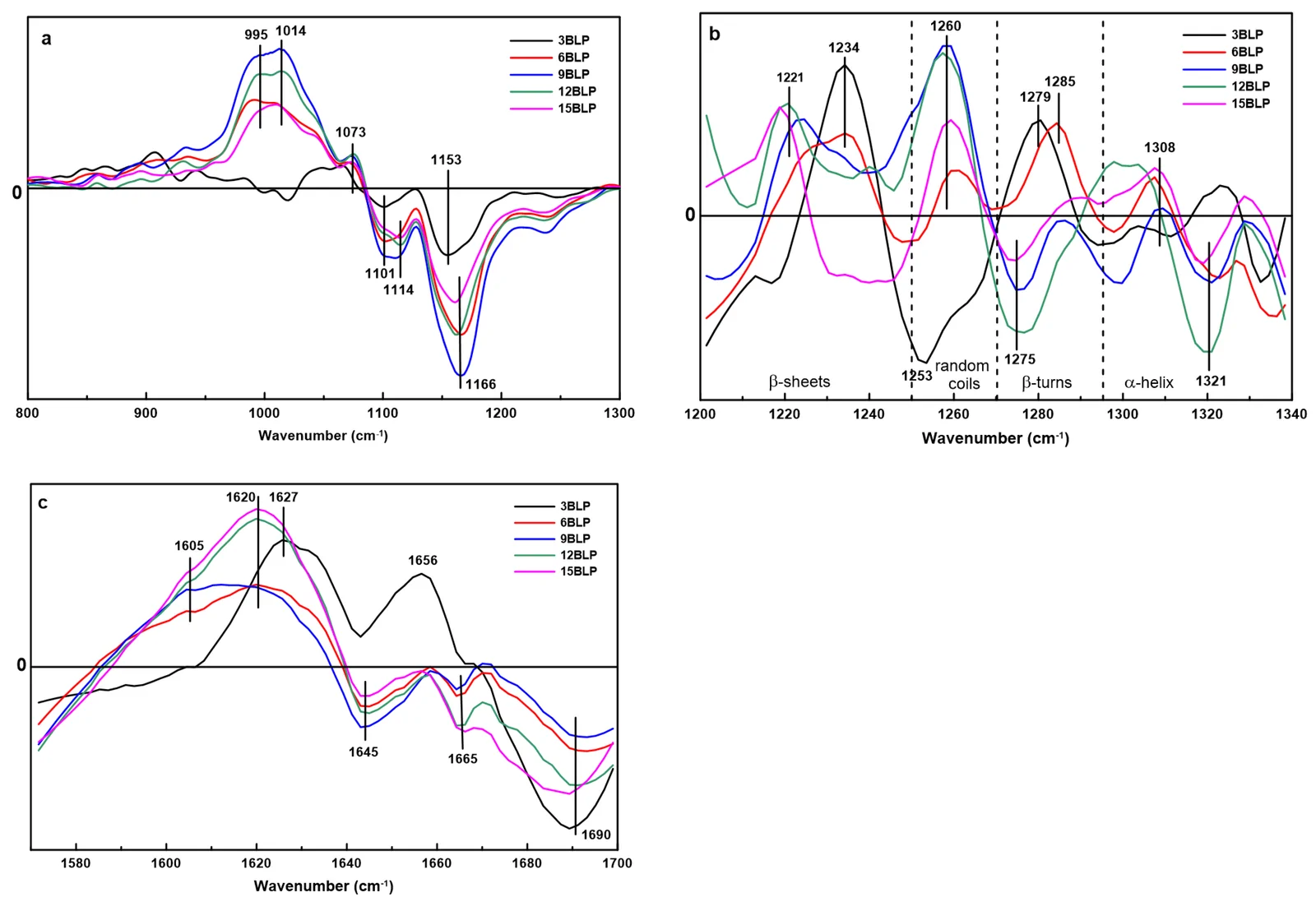
FT-IR difference spectra calculated in three spectral ranges: (**a**) 800–1300 cm^−1^, (**b**) 1200–1340 cm^−1^ (amide III band), and (**c**) 1570–1700 cm^−1^ (amide I band) for cookies enriches with black lime powder (BLP) in the amount 3%, 6%, 9%, 12%, and 15% (3BLP, 6BLP, 9BLP, 12BLP, and 15BLP).

To determine the effect of BLP on the protein and polysaccharide structure in the cookies, the FT-IR difference spectra in three spectral ranges were calculated. The difference spectra are presented in the Figure 1. The first spectral range (800–1300 cm^−1^) (Figure 1a) may be assigned to skeletal vibrations in polysaccharides [58]. The difference spectrum shows positive bands at 995, 1014 and 1073 cm^−1^, and negative bands at 1101, 1114, 1153 and 1166 cm^−1^. Intensity of the band at 1014 cm^−1^ increases with an increase of the lime powder amount in the cookies. A similar band is visible in the spectrum of BLP (Figure S1 in the Supplementary Material). Hence it can be concluded that the band at 1014 cm^−1^ is related with the skeletal vibrations of polysaccharides in lime powder. The band at 995 cm^−1^ may be assigned to skeletal oscillations in wheat starch and its crystalline form [43]. Intensity of this band also increases with an increase in the amount of BLP to 12% in the cookies. Spectrum 15BLP shows only band at 1014 cm^−1^. The intensity increase (band at 995 cm^−1^) may indicate that starch becomes more crystalline after lime powder addition. It is a well-known fact that the starch crystallinity and molecular organization can be affected by water availability [44]. It can be connected with competition for water between basic components of cookies’ dough (wheat starch and wheat gluten) and polysaccharides present in the BLP. The lime powder is rich in the dietary fibre (Table 1). Although the moisture content and water activity of the cookies increased with BLP addition, these bulk parameters describe the final product and do not necessarily reflect the local availability of water to starch during baking. Hydrophilic dietary fibre present in BLP may retain part of the water within fibre-rich domains, thereby modifying its spatial distribution and reducing the amount of water locally available for starch swelling and gelatinization. Thus, a higher overall moisture content may coexist with locally restricted water availability in starch-rich regions during baking, which could favour the preservation of more ordered starch structures. Similar competition for water between basic wheat dough components and dietary fibre preparations has been observed by Miś et al. [42], and Gomez et al. [59]. Moreover, a weak negative band located at 1101 cm^−1^ was shifted to 1114 cm^−1^ as a result of 12% and 15% addition of BLP. A shift to 1166 cm^−1^ (6BLP–15BLP) was also observed in the case of band at 1153 cm^−1^ (3BLP). The bands at 1101 and 1153 cm^−1^ are characteristic for the raw wheat starch that can be assigned to C-O, C-C and C-O-H stretching, and bending and asymmetric stretching of the C-O-C glycosidic bond [44]. The shift of these two bands toward longer wavenumbers and negative orientation can be connected with the baking process and interactions between wheat starch and polysaccharidic components of the lime powder. Disappearance of these bands was observed by other authors as a result of heat treatment of the starch-inulin and starch-cellulose mixtures [44].

Amide I and amide III bands are used to determine changes in the secondary structure of proteins (in this case wheat gluten). Figure 1b presents difference spectra calculated in the amide III band. Using the lime powder concentration higher than 3% caused considerable changes in the β-sheets region i.e., shift of the band at 1234 cm^−1^ to 1221 cm^−1^. The shift may indicate formation of the hydrogen bonds of II type. The hydrogen bonds can be formed between wheat gluten polypeptide chains and polysaccharides from the BLP. The hydrogen bonds of II type were observed by Nawrocka et al. [45]. Additionally, analysis of difference spectra showed a positive band at 1260 cm^−1^ (random coils), and negative bands at 1275 cm^−1^ (β-turns) and 1321 cm^−1^ (α-helices). Presence of these bands suggests disaggregation of α-helices and β-turns into random coils. The difference spectra in the amide I band also showed other changes in the gluten structure in the case of using 6–15% of BLP (Figure 1c). The positive bands assigned to aggregates and structured aggregates (pseudo-β-sheets) were located at 1605 and 1620 cm^−1^, while negative bands connected with β-turns with intra- and intermolecular hydrogen bonds and antiparallel β-sheets were observed at 1645, 1665 and 1690 cm^−1^, respectively. Analysis of both amide bands may indicate that addition of the lime powder to cookies may cause disaggregation of the basic secondary structures (β-sheets, β-turns and α-helices) into aggregates and random coils.

### 2.8. Sensory Evaluation of Cookies

**Figure 2:**
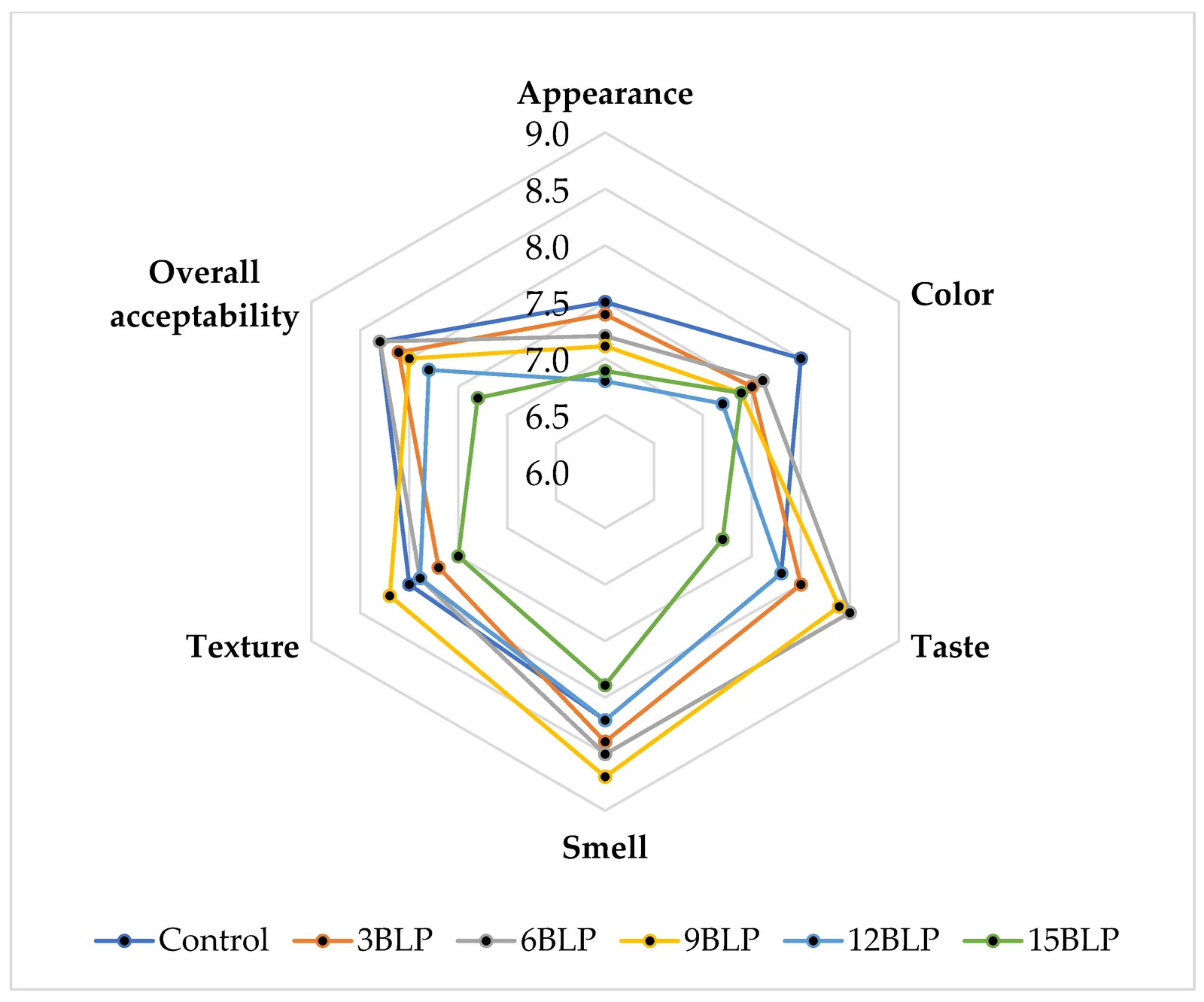
Sensory assessment of control and BLP-enriched cookies (*n* = 46). Values are mean scores on a 9-point hedonic scale. Control, cookies without BLP; 3BLP–15BLP, cookies in which 3–15% of wheat flour was replaced with BLP (black lime powder). Full descriptive statistics are provided in Table S1, and statistical groupings are provided in Table S2.

Overall acceptability remained statistically comparable with that of the control at wheat-flour replacement levels of 3–12%, whereas 15BLP received a significantly lower score (Figure 2; Tables S1 and S2 in the Supplementary Material). Texture followed the same pattern, with a significant decrease only at 15% BLP. Cookies containing 3%, 6%, or 9% BLP received higher mean smell and taste scores than the control. The highest mean smell score occurred in 9BLP, while 6BLP had the highest mean taste score and shared the highest overall-acceptability score with the control. Appearance scores decreased significantly at 12% and 15% replacement, and color scores decreased significantly from 9% onward (Table S2). These sensory responses are consistent with the objective color measurements: increasing BLP produced progressive darkening and larger ΔE values (Table 5). Descriptive statistics in Table S1 show that most sensory ratings remained in the upper portion of the 9-point hedonic scale, with medians commonly between 7 and 9. Folorunso et al. reported that cookies containing 10% lemon-, tangerine-, or orange-peel powder had overall-acceptability scores equal to or higher than those of the control [60]. Their formulations also contained date powder as a sugar replacer, however, so the sensory contribution of citrus peel cannot be isolated. Al-Saab and Gadallah found that 5% orange-peel powder maintained overall acceptability, whereas 10–20% reduced it [57]. Differences among studies may reflect the distinct flavor, color, composition, and processing of each citrus ingredient. Under the conditions of the present study, 12% was the highest BLP replacement level that maintained overall acceptability comparable with the control, although appearance and color were already affected at that concentration. The 6–9% range provided the most favorable sensory balance: 6BLP achieved the highest mean taste and joint-highest overall-acceptability scores, whereas 9BLP achieved the highest mean smell score. Accordingly, the preferred formulation depends on whether taste, aroma, appearance, or maximal fiber enrichment is prioritized.

## 3. Materials and Methods

### 3.1. Chemicals and Enzymes

Folin–Ciocalteu reagent, 2,2′-azino-bis(3-ethylbenzothiazoline-6-sulfonic acid) (ABTS), 2,2-diphenyl-1-picrylhydrazyl (DPPH), Trolox, methanol, ethanol, hexane, acetonitrile, formic acid, sodium carbonate, potassium persulfate, and Tris-HCl were used. Analytical standards for chromatographic analysis included gallic acid, neochlorogenic acid (3-O-caffeoylquinic acid), protocatechuic acid, chlorogenic acid (5-O-caffeoylquinic acid), cryptochlorogenic acid (4-O-caffeoylquinic acid), 4-hydroxybenzoic acid, caffeic acid, syringic acid, *p*-coumaric acid (4-hydroxycinnamic acid), ferulic acid, and salicylic acid. Unless otherwise stated, chemical reagents and analytical standards were purchased from Sigma-Aldrich (Poznań, Poland). Heat-stable α-amylase, protease, and amyloglucosidase used for dietary-fiber determination were supplied by Megazyme International Ireland Ltd. (Wicklow, Ireland).

### 3.2. Raw Materials and Preparation of BLP

Dried black lime (*Citrus × aurantiifolia*) fruits of Iraqi origin were purchased from a local food supplier (Baghdad, Iraq). According to the information provided by the supplier, the fruits were briefly blanched in salted water and subsequently sun-dried until they became hard and dark brown to black. The whole fruits were ground in a GRINDOMIX GM 200 knife mill (Retsch GmbH, Haan, Germany) and passed through a sieve with a 300 µm aperture. The resulting material was designated black lime powder (BLP). Additional ingredients were commercial coarse-milled type 500 wheat flour (Krupczatka, Lubella, Lublin, Poland), granulated white sugar (Polski Cukier, Krajowa Grupa Spożywcza S.A., Toruń, Poland), butter containing 82% milk fat (OSM Krasnystaw, Krasnystaw, Poland), and fresh size-M hen eggs (Fermy Wiejak, Eggs Investment Sp. z o.o., Mełgiew, Poland). All ingredients were purchased from local retailers in Lublin, Poland.

### 3.3. Cookie Preparation

Shortbread cookies were produced by replacing wheat flour with BLP on a weight-for-weight basis at 3%, 6%, 9%, 12%, or 15%. The resulting formulations were designated 3BLP, 6BLP, 9BLP, 12BLP, and 15BLP, respectively. Cookies prepared without BLP served as the control. The detailed formulations of the control and BLP-enriched cookies are provided in Table S3 in the Supplementary Materials. Wheat flour, sugar, and the appropriate amount of BLP were blended for 5 min in a KitchenAid Heavy Duty mixer (T-5KPM5EER; KitchenAid, Benton Harbor, MI, USA). Butter was added, and mixing continued until a crumbly consistency was obtained. An egg was then incorporated, and the dough was mixed at low speed for 5 min. The dough was rolled to a thickness of 6 mm and cut manually into 40 mm diameter discs with a stainless-steel cutter. Cookies were baked in a convection oven (CMP 61; Rational, Landsberg am Lech, Germany) at 200 °C for 15 min and cooled to 21 °C before analysis.

### 3.4. Proximate Composition

Moisture, protein, fat, ash, soluble dietary fiber, and insoluble dietary fiber were determined in wheat flour, BLP, and cookies using the procedures described by Krawęcka et al. [61]. Total dietary fiber was calculated as the sum of the soluble and insoluble fractions. Available carbohydrate content was calculated by difference on an as-is basis as 100 − (moisture + protein + fat + ash + total dietary fiber) and was subsequently converted to a dry-matter basis for reporting.

### 3.5. Preparation of Extracts for TPC and Antioxidant Assays

Aqueous methanol extracts were prepared using a procedure adapted from Pérez-Jiménez and Saura-Calixto [62]. A portion of wheat flour, BLP, or ground cookie corresponding to 0.500 g of dry matter was mixed with 5.0 mL of methanol/water (50:50, *v*/*v*; pH 2.0) and shaken at 200 rpm for 60 min at room temperature. The suspension was centrifuged at 5000× *g* for 10 min at 4 °C. The supernatant was brought to a final volume of 10.0 mL and protected from light until analysis.

Spectrophotometric measurements were performed using a Helios Gamma UV–Vis spectrophotometer (Thermo Spectronic, Cambridge, UK).

### 3.6. Antioxidant Activity

#### 3.6.1. ABTS Assay

The ABTS radical-cation decolorization assay was performed according to Re et al. [63], with modifications. The radical cation was generated by mixing 7.0 mM ABTS with 2.45 mM potassium persulfate and allowing the mixture to react for 16 h in the dark. Before analysis, the solution was diluted with ethanol to an absorbance of 0.700 ± 0.020 at 734 nm. Sample extract (30 µL) was combined with 3.00 mL of the ABTS working solution, incubated for 6 min at 25 °C in the dark, and measured at 734 nm.

#### 3.6.2. DPPH Assay

The DPPH assay was performed according to Shimamura et al. [64], with modifications. A 0.2 mM DPPH solution in ethanol was adjusted to a control absorbance of 1.00 ± 0.05 at 517 nm. Sample extract (200 µL) was mixed with 800 µL of 0.1 M Tris-HCl buffer (pH 7.4) and 1.00 mL of DPPH solution. After 30 min of incubation in the dark, absorbance was measured at 517 nm. Appropriate reagent blanks and controls were included in both assays. Radical-scavenging activity was calculated from the decrease in absorbance relative to the corresponding control. Separate Trolox calibration curves were prepared for ABTS (0–1.50 mM) and DPPH (0–0.30 mM). The Trolox-equivalent concentration was converted to antioxidant activity using the final extract volume, sample dry mass, and any applied dilution. Results were expressed as µmol Trolox equivalents per gram of dry matter (µmol TE/g DW).

### 3.7. Total Phenolic Content

Total phenolic content (TPC) was determined using the Folin–Ciocalteu assay [65]. Extract (0.5 mL) was mixed with 0.5 mL of distilled water, 2.0 mL of Folin–Ciocalteu reagent diluted 1:5 (*v*/*v*) with water, and 2.0 mL of 10% (*w*/*v*) Na_2_CO_3_. The mixture was incubated for 60 min, after which absorbance was measured at 720 nm against a 50% methanol blank. A 10-level gallic-acid calibration curve covering 0–1 mg mL^−1^ was used (R^2^ = 0.997). Results were expressed as milligrams of gallic acid equivalents per gram of dry matter (mg GAE/g DW).

### 3.8. LC-ESI-MS/MS Analysis of Phenolic Compounds

Phenolic acids and flavonoids in BLP were analyzed by high-performance liquid chromatography coupled with electrospray-ionization tandem mass spectrometry (LC-ESI-MS/MS), using a modified procedure based on Łubek-Nguyen et al. [66]. An Agilent 1200 Series HPLC system (Agilent Technologies, Santa Clara, CA, USA) coupled to a 3200 QTRAP tandem mass spectrometer (AB Sciex, Redwood City, -CA, USA) equipped with an electrospray ionization (ESI) source was used for the analyses. The chromatographic and mass-spectrometric systems were controlled using Analyst 1.5 software (AB Sciex, Redwood City, MA, USA), which was also used for data acquisition and processing. A two-step sequential extraction was applied. BLP was first extracted with 80% methanol (*v*/*v*). After separation of the first extract, the residual material was extracted with 50% methanol (*v*/*v*). The two fractions were retained separately and analyzed independently. Chromatographic separation was performed at 25 °C on an Eclipse XDB-C18 column (4.6 × 150 mm, 5.0 µm; Agilent Technologies, Santa Clara, CA, USA). Mobile phase A was 0.1% formic acid in water, and mobile phase B was acetonitrile containing 0.1% formic acid. The injection volume was 3 µL, and the flow rate was 400 µL min^−1^. The gradient program was as follows: 0–1.5 min, 13% B; 1.5–2 min, 13–20% B; 2–4.5 min, 20% B; 4.5–5 min, 20–25% B; 5–8 min, 25% B; 8–9 min, 25–33% B; 9–11 min, 33% B; 11–13 min, 33–60% B; 13–16 min, 60% B; 16–18 min, 60–80% B; 18–21 min, 80% B; and 21–28 min, 13% B. Mass-spectrometric detection was performed using electrospray ionization in negative-ion mode. The operating conditions were a capillary temperature of 500 °C, curtain-gas pressure of 30 psi, nebulizer-gas pressure of 55 psi, and ion-source voltage of −4500 V. Nitrogen was used as the curtain and collision gas. Quantitative analyses were performed in multiple-reaction-monitoring (MRM) mode. The precursor-to-product ion transitions and collision energy (CE) values were optimized individually for each analyte and MRM transition. Each standard solution and sample extract was injected in triplicate. Limits of detection (LOD) and quantification (LOQ) were established at signal-to-noise ratios of 5:1 and 10:1, respectively. Compounds were identified by comparing their retention times and MS/MS transitions with those of authentic standards. Tracked MRM transitions and collision energies followed those reported by Łubek-Nguyen et al. [66]. Quantification of the identified metabolites was performed using calibration curves generated in MRM mode based on the most intense transition for each analyte (Table S4 in the Supplementary Material). Signals below the LOD were reported as not detected (ND), whereas detected signals below the LOQ were reported as below the quantification limit (BQL).

### 3.9. Color Measurement

The color of wheat flour, BLP, and cookies was measured with a handheld sphere spectrophotometer (Ci64; X-Rite Incorporated, Grand Rapids, MI, USA) and expressed in the CIELAB color space. L* represented lightness, a* the green–red coordinate, and b* the blue–yellow coordinate. Five measurements were performed for each sample. For cookies, total color difference (ΔE) was calculated relative to the control formulation.

### 3.10. Moisture Content and Water Activity

Water activity was measured in 2 g portions of ground cookie at 22 °C using a LabMaster instrument (Novasina AG, Lachen, Switzerland). Moisture content was determined gravimetrically by drying cookie samples at 105 °C in a laboratory oven (SLN 53 STD/TOP+; POL-EKO-APARATURA, Wodzisław Śląski, Poland) to constant mass. Wet-basis moisture content was calculated as the mass lost during drying relative to the initial sample mass [65].

### 3.11. Spread Ratio, Volume Expansion, and Baking Loss

The diameter and thickness of the cookies were measured after cooling to room temperature using a digital caliper (LUX-TOOLS, 150 mm; OBI Group Sourcing GmbH, Wermelskirchen, Germany). The results of these measurements are presented in the Supplementary Material (Table S5). The spread ratio was calculated as the ratio of cookie diameter to thickness.

Baking loss was determined from the percentage difference between the mass of the dough before baking and the mass of the cookies after baking and cooling, according to the method described by Dadalı and Özcan [67].

Cookie volume was calculated assuming cylindrical geometry based on diameter and thickness. The initial dough volume was calculated from the nominal dimensions of the dough discs (40 mm diameter and 6 mm thickness), while the volume after baking was calculated using the measured dimensions of the cooled cookies. Volume expansion was expressed as the percentage change in calculated volume relative to the initial dough volume.

### 3.12. Cutting Force

Cookie texture was assessed using a Zwick universal testing machine (Z020/TN2S; Zwick, Ulm, Germany), following the cutting-test approach described by Krajewska and Dziki [68]. The maximum force recorded during cutting was expressed in newtons and used as an instrumental measure of cookie fracture resistance. The samples were positioned centrally beneath the cutting blade. Individual cookies were cut using a 1 mm thick blade at a crosshead speed of 20 mm min^−1^ until the gap between the blade and the platform reached 0.1 mm.

### 3.13. FTIR Spectroscopy

Fourier-transform infrared (FTIR) spectra were recorded with a Nicolet 6700 spectrometer equipped with a diamond attenuated-total-reflectance accessory (Thermo Scientific, Madison, WI, USA), following Kłosok et al. [69]. Powdered samples were scanned from 400 to 4000 cm^−1^ at a resolution of 4 cm^−1^. Five spectra were recorded for each sample and averaged. Spectra were processed in OriginPro 9.0 (OriginLab Corporation, Northampton, MA, USA) using the approach described by Nawrocka et al. [70]. All spectra were area-normalized. Difference spectra were calculated by subtracting the control-cookie spectrum from each BLP-enriched cookie spectrum in three regions: 800–1300 cm^−1^, 1200–1340 cm^−1^ (amide III), and 1570–1700 cm^−1^ (amide I). Assignments used to interpret changes in gluten secondary structure in the amide I and amide III regions followed Nawrocka et al. [71] and Stani et al. [72], respectively.

### 3.14. Sensory Evaluation

Consumer acceptability was evaluated by a panel of 46 untrained adult participants recruited from the University of Life Sciences in Lublin. The panel consisted of 24 women and 22 men, aged 21–45 years. Participants were recruited on a voluntary basis according to the following inclusion criteria: age ≥ 18 years, regular consumption of cookies, and willingness to participate in the sensory evaluation. Individuals reporting food allergies or intolerances to any of the ingredients used in the tested formulations, as well as those with conditions that could impair taste or olfactory perception, were excluded from the study. None of the participants had received formal sensory analysis training prior to the evaluation. Consumer acceptability was assessed using a nine-point hedonic scale, where 1 corresponded to “dislike very much” and 9 to “like very much.” The evaluated sensory attributes included appearance, color, aroma, taste, texture, and overall acceptability of each formulation. The assessment was carried out under daylight conditions in a room maintained at 20 °C. Each participant evaluated all samples, which were presented in a randomized order and identified using three-digit random codes. Participants were instructed to rinse their mouths with water between samples to minimize potential carry-over effects. Prior to the sensory evaluation, all participants were informed about the purpose of the study, the study procedures, and the voluntary nature of their participation. Informed consent was obtained from all participants before the evaluation. Participants were informed that they could withdraw from the study at any time without providing a reason. Appropriate measures were taken to ensure participant safety, privacy, and confidentiality. Approval by a bioethics committee was not required because the study involved only the non-invasive sensory assessment of food products (cookies) by adult participants using a nine-point hedonic scale. No medical, diagnostic, or therapeutic procedures were performed, no biological material was collected, and no health-related outcomes were assessed. Therefore, the sensory evaluation was not considered a medical experiment requiring prior approval by a bioethics committee. The study was conducted in accordance with the principles of the Declaration of Helsinki.

### 3.15. Statistical Analysis of Data

Statistical analysis of the data was conducted using Statistica 13.3 (TIBCO Software, Palo Alto, CA, USA). Differences among samples for physicochemical and other instrumental measurements were evaluated by one-way analysis of variance (ANOVA), followed by Tukey’s post hoc test. Pearson’s correlation coefficient was used to assess relationships between the analyzed variables. For sensory evaluation of the cookies, differences among the control and black lime powder-substituted samples were assessed using the Friedman test, and when statistically significant differences were identified, post hoc comparisons were performed. Statistical significance was set at *p* < 0.05. All instrumental measurements were performed in at least three replicates.

## 4. Conclusions

BLP was compositionally distinct from wheat flour and represented a rich source of dietary fiber, minerals, and phenolic compounds, including protocatechuic, *p*-coumaric, vanillic, and ferulic acids as well as the flavone C-glycosides isovitexin and vitexin. Its incorporation into shortbread cookies resulted in concentration-dependent increases in dietary fiber, TPC, and radical-scavenging activity, while also affecting moisture, water activity, cutting force, color, and FTIR-derived structural features.

Sensory acceptance was strongly dependent on the level of BLP incorporation. The 6–9% BLP range provided the most favorable balance between compositional enrichment and sensory quality, whereas higher levels produced more pronounced changes in appearance, flavor, and texture. Thus, the main technological challenge is to maximize the nutritional and functional benefits of BLP while limiting undesirable quality changes. Further optimization of BLP level, formulation, particle characteristics, and processing conditions may help to improve this balance.

Overall, the results indicate that BLP has potential as a fiber- and phenolic-rich ingredient for shortbread cookies and other baked cereal products. Although the low water activity of the cookies (<0.44) suggests a low risk of microbial spoilage, it does not by itself confirm extended shelf-life. In particular, oxidative stability during storage remains to be evaluated because of the relatively high fat content and the complex polyphenol–lipid matrix. Storage stability and individual phenolic compounds in the cookies were not evaluated in this study. Future studies should therefore assess lipid oxidation during storage and the retention or transformation of individual phenolic compounds during baking using targeted LC-MS/MS analysis.

## Figures and Tables

**Table 1:** Proximate composition, total phenolic content, antioxidant activity, and color parameters of BLP and wheat flour.

Parameter	BLP	WFL
Protein [% DW]	5.15 ± 0.09 ^b^	9.93 ± 0.07 ^a^
Fat [% DW]	2.39 ± 0.03 ^a^	1.44 ± 0.04 ^b^
Ash [% DW]	5.10 ± 0.03 ^a^	0.48 ± 0.01 ^b^
Available carbohydrates [% DW]	33.62 ± 0.65 ^b^	85.97 ± 0.03 ^a^
Total fiber [% DW]	53.75 ± 0.56 ^a^	2.17 ± 0.07 ^b^
Insoluble fiber [% DW]	29.78 ± 0.51 ^a^	1.19 ± 0.06 ^b^
Soluble fiber [% DW]	23.97 ± 0.14 ^a^	0.99 ± 0.04 ^b^
TPC [mg GAE/g DW]	21.89 ± 0.43 ^a^	0.83 ± 0.01 ^b^
ABTS [µmol TE/g DW]	17.89 ± 1.06 ^a^	0.46 ± 0.02 ^b^
DPPH [µmol TE/g DW]	29.29 ± 0.66 ^a^	0.38 ± 0.01 ^b^
L*	28.04 ± 0.47 ^b^	90.80 ± 0.28 ^a^
a*	6.99 ± 0.20 ^a^	2.22 ± 0.06 ^b^
b*	9.17 ± 0.40 ^b^	15.04 ± 0.42 ^a^

Values are expressed as mean ± standard deviation (*n* = 3). Different superscript letters within the same row indicate significant differences between samples (*p* < 0.05). BLP—black lime powder; WFL—wheat flour; TPC—total phenolic content; GAE—gallic acid equivalents; ABTS—2,2′-azino-bis(3-ethylbenzothiazoline-6-sulfonic acid) radical cation scavenging activity; DPPH—2,2-diphenyl-1-picrylhydrazyl radical scavenging activity; DW—dry weight; TE—Trolox equivalents; L*—lightness; a*—green–red coordinate; b*—blue–yellow coordinate.

**Table 2:** Content of phenolic acids, flavonoid aglycones, and flavonoid glycosides in fractions by two-step sequential extraction of black lime powder with 80% MeOH and 50% MeOH, expressed as µg/g of sample dry weight.

Compound	80% MeOH	50% MeOH	Total E1 + E2
Phenolic acids			
Gallic acid	ND	0.829 ± 0.003	0.829 ± 0.003
Protocatechuic acid	262.080 ± 2.429	30.761 ± 0.117	292.841 ± 2.311
5-Caffeoylquinic acid	ND	BQL	BQL
4-Hydroxybenzoic acid	11.607 ± 0.185	BQL	11.607 ± 0.185
Gentisic acid	14.674 ± 0.418	0.820 ± 0.054	15.494 ± 0.377
Vanillic acid	166.632 ± 26.435	BQL	166.632 ± 26.435
Syringic acid	12.958 ± 0.367	ND	12.958 ± 0.367
3-Hydroxybenzoic acid	BQL	ND	BQL
*p*-Coumaric acid	160.888 ± 3.906	10.653 ± 0.302	171.541 ± 4.163
Sinapic acid	16.760 ± 0.498	2.159 ± 0.124	18.919 ± 0.495
Ferulic acid	127.563 ± 2.525	ND	127.563 ± 2.525
Salicylic acid	BQL	ND	BQL
Flavonoid aglycones			
Luteolin	0.320 ± 0.003	0.059 ± 0.002	0.380 ± 0.004
Eriodictyol	0.017 ± 0.001	ND	0.017 ± 0.001
Quercetin	0.759 ± 0.017	0.146 ± 0.003	0.905 ± 0.016
Apigenin	1.720 ± 0.077	0.130 ± 0.006	1.850 ± 0.077
Isorhamnetin	8.615 ± 0.185	0.959 ± 0.016	9.574 ± 0.187
Flavonoid glycosides			
Eriocitrin	0.689 ± 0.055	3.752 ± 0.074	4.441 ± 0.122
Isovitexin + vitexin	143.579 ± 5.955	17.719 ± 0.658	161.298 ± 5.428
Luteoloside	5.330 ± 0.202	1.429 ± 0.054	6.759 ± 0.183
Eriodictyol-7-glucopyranoside	BQL	BQL	BQL
Narcissoside	17.278 ± 0.398	3.346 ± 0.097	20.625 ± 0.302
Narirutin	BQL	3.870 ± 0.041	3.870 ± 0.041
Isorhamnetin 3-O-glucoside	BQL	BQL	BQL
Apigenin 7-O-glucoside	BQL	BQL	BQL
Naringenin 7-O-glucoside	0.823 ± 0.028	ND	0.823 ± 0.028

Values are presented as mean ± SD (*n* = 3) and expressed as µg/g of sample dry weight (DW). The results obtained for each extract were recalculated to the initial sample dry matter by accounting for the extraction yield of the respective fraction. Total (E1 + E2) represents the sum of the recalculated quantifiable contents obtained in the two sequential extraction steps. ND—not detected; BQL—detected but below the limit of quantification.

**Table 3:** Physical and technological properties of control and BLP-enriched cookies.

Sample	Moisture Content [% Wet Basis]	Water Activity	Cutting Force [N]	Spread Ratio	Volume Expansion [%]	Baking Loos [%]
Control	3.799 ± 0.049 ^c^	0.293 ± 0.006 ^d^	52.91 ± 2.16 ^a^	5.48 ± 0.17 ^abc^	71.20 ± 6.83 ^ab^	12.46 ± 0.10 ^a^
3BLP	4.067 ± 0.005 ^b^	0.415 ± 0.007 ^c^	45.83 ± 2.09 ^b^	5.32 ± 0.16 ^c^	72.46 ± 7.05 ^a^	12.29 ± 0.08 ^ab^
6BLP	4.080 ± 0.050 ^ab^	0.417 ± 0.001 ^bc^	44.29 ± 0.83 ^bc^	5.37 ± 0.22 ^bc^	69.82 ± 6.72 ^ab^	12.27 ± 0.06 ^ab^
9BLP	4.088 ± 0.026 ^ab^	0.420 ± 0.003 ^bc^	39.58 ± 1.47 ^cd^	5.49 ± 0.18 ^abc^	65.27 ± 6.46 ^ab^	12.14 ± 0.10 ^b^
12BLP	4.209 ± 0.081 ^a^	0.428 ± 0.002 ^ab^	35.45 ± 2.02 ^de^	5.59 ± 0.14 ^ab^	64.28 ± 4.85 ^ab^	12.10 ± 0.06 ^b^
15BLP	4.198 ± 0.050 ^ab^	0.433 ± 0.002 ^a^	32.77 ± 2.25 ^e^	5.63 ± 0.16 ^a^	62.73 ± 7.50 ^b^	12.11 ± 0.04 ^b^

Values are expressed as mean ± standard deviation. Different superscript letters within the same column indicate significant differences between samples (*p* < 0.05). For moisture content, water activity, and cutting force, *n* = 6; for spread ratio and volume expansion, *n* = 10; and for baking loss, *n* = 3. Control—cookies without BLP; 3BLP–15BLP—cookies in which 3–15% of wheat flour was replaced with BLP (black lime powder).

**Table 4:** Proximate composition of control and BLP-enriched cookies (% DW).

Sample	Protein [%]	Fat [%]	Ash [%]	SF [%]	IF [%]	Total Fiber [%]	AC [%]
Control	7.66 ± 0.02 ^a^	30.04 ± 0.08 ^a^	0.72 ± 0.02 ^f^	0.62 ± 0.01 ^f^	2.04 ± 0.05 ^f^	2.67 ± 0.04 ^f^	58.91
3BLP	7.58 ± 0.04 ^a^	29.90 ± 0.31 ^a^	0.82 ± 0.02 ^e^	1.29 ± 0.08 ^e^	2.91 ± 0.06 ^e^	4.20 ± 0.14 ^e^	57.50
6BLP	7.48 ± 0.04 ^b^	29.77 ± 0.62 ^a^	0.97 ± 0.01 ^d^	2.12 ± 0.06 ^d^	3.76 ± 0.08 ^d^	5.87 ± 0.10 ^d^	55.91
9BLP	7.40 ± 0.02 ^bc^	30.08 ± 0.10 ^a^	1.12 ± 0.03 ^c^	2.74 ± 0.06 ^c^	4.60 ± 0.02 ^c^	7.34 ± 0.07 ^c^	54.10
12BLP	7.35 ± 0.02 ^c^	30.08 ± 0.07 ^a^	1.21 ± 0.02 ^b^	3.47 ± 0.05 ^b^	5.43 ± 0.08 ^b^	8.90 ± 0.11 ^b^	52.46
15BLP	7.22 ± 0.06 ^d^	30.42 ± 0.57 ^a^	1.36 ± 0.02 ^a^	4.18 ± 0.03 ^a^	6.35 ± 0.07 ^a^	10.53 ± 0.10 ^a^	50.47

Values are expressed as mean ± standard deviation (*n* = 3). Different superscript letters within the same column indicate significant differences between samples (*p* < 0.05). Control—cookies without BLP; 3BLP–15BLP—cookies in which 3–15% of wheat flour was replaced with BLP (black lime powder); SF—soluble fiber; IF—insoluble fiber; AC—available carbohydrates.

**Table 5:** Color parameters and total color difference of control and BLP-enriched cookies.

Sample	L*	a*	b*	ΔE
Control	73.22 ± 1.45 ^a^	6.77 ± 1.71 ^a^	28.89 ± 2.33 ^a^	–
3BLP	56.05 ± 2.44 ^b^	5.91 ± 0.37 ^a^	16.89 ± 1.62 ^b^	20.97
6BLP	53.89 ± 2.38 ^bc^	5.57 ± 0.33 ^a^	14.32 ± 0.66 ^bc^	24.24
9BLP	50.08 ± 1.80 ^cd^	5.43 ± 0.47 ^a^	15.38 ± 0.71 ^bc^	26.84
12BLP	47.22 ± 4.35 ^de^	6.71 ± 0.72 ^a^	15.04 ± 2.12 ^bc^	29.46
15BLP	42.69 ± 1.63 ^e^	6.15 ± 0.90 ^a^	12.75 ± 0.83 ^c^	34.61

Values are expressed as mean ± standard deviation. Different superscript letters within the same column indicate significant differences between samples (*p* < 0.05). L*—lightness; a*—green–red coordinate; b*—blue–yellow coordinate; ΔE—total color difference relative to the control; Control—cookies without BLP; 3BLP–15BLP—cookies in which 3–15% of wheat flour was replaced with BLP (black lime powder).

**Table 6:** Total phenolic content and antioxidant activity of control and BLP-enriched cookies.

Sample	TPC [mg GAE/g DW]	ABTS [µmol TE/g DW]	DPPH [µmol TE/g DW]
Control	0.69 ± 0.03 ^e^	0.30 ± 0.07 ^e^	0.13 ± 0.02 ^f^
3BLP	1.30 ± 0.11 ^d^	0.74 ± 0.10 ^d^	0.88 ± 0.15 ^e^
6BLP	1.65 ± 0.09 ^d^	1.33 ± 0.14 ^c^	1.49 ± 0.17 ^d^
9BLP	2.36 ± 0.16 ^c^	2.14 ± 0.20 ^b^	2.55 ± 0.34 ^c^
12BLP	2.96 ± 0.22 ^b^	2.42 ± 0.17 ^b^	3.14 ± 0.20 ^b^
15BLP	3.78 ± 0.25 ^a^	3.33 ± 0.22 ^a^	3.76 ± 0.22 ^a^

Values are expressed as mean ± standard deviation. Different superscript letters within the same column indicate significant differences between samples (*p* < 0.05). ABTS—2,2′-azino-bis(3-ethylbenzothiazoline-6-sulfonic acid) radical cation scavenging activity; DPPH—2,2-diphenyl-1-picrylhydrazyl radical scavenging activity; BLP—black lime powder; TPC—total phenolic content; DW—dry weight; GAE—gallic acid equivalents; TE—Trolox equivalents; Control—cookies without BLP; 3BLP–15BLP—cookies in which 3–15% of wheat flour was replaced with BLP (black lime powder).

## Data Availability

The data presented in this study are available from the corresponding authors upon reasonable request.

## Supplementary Materials

The following supporting information can be downloaded at: https://www.mdpi.com/article/10.3390/molecules31183271/s1, Figure S1: FTIR spectra of black lime powder in two spectral ranges: 800–1350 cm^−1^ and 1500–1800 cm^−1^, Table S1: Descriptive statistics of sensory evaluation of control and black lime powder-substituted samples (*n* = 46), Table S2: Statistical groupings for sensory evaluation of control and black lime-enriched samples, Table S3: Formulation of control and BLP-enriched shortbread cookies (% *w*/*w* of total dough), Table S4: Analytical parameters used for quantitative LC-MS/MS analysis of polyphenols, Table S5: Thickness and diameter of shortbread cookies.

## Abbreviations

The following abbreviations are used in this manuscript:

ABTS	2,2′-azino-bis(3-ethylbenzothiazoline-6-sulfonic acid)
ANOVA	analysis of variance
BLP	black lime powder
DPPH	2,2-diphenyl-1-picrylhydrazyl
DW	dry weight
FTIR	Fourier-transform infrared spectroscopy
GAE	gallic acid equivalents
HPLC	high-performance liquid chromatography
LC–ESI–MS/MS	liquid chromatography–electrospray ionization tandem mass spectrometry
LOD	limit of detection
LOQ	limit of quantification
MRM	multiple reaction monitoring
TE	Trolox equivalents
TPC	total phenolic content

## References

[1] Marcìa-Fuentes J.A., Aleman R.S., Areche F.O., Flores D.C., Roman A.V., Martín-Vertedor D., Montero-Fernández I. (2026). Functional foods: A review of foods ingredient and their health benefits. Food Humanit..

[2] Fekete M., Lehoczki A., Kryczyk-Poprawa A., Zábó V., Varga J.T., Bálint M., Fazekas-Pongor V., Csípő T., Rząsa-Duran E., Varga P. (2025). Functional Foods in Modern Nutrition Science: Mechanisms, Evidence, and Public Health Implications. Nutrients.

[3] Raczkowska E., Wojdyło A., Nowicka P. (2024). The use of blackcurrant pomace and erythritol to optimise the functional properties of shortbread cookies. Sci. Rep..

[4] Raczkowska E., Wojdyło A., Nowicka P. (2024). Effect of the Addition of Apple Pomace and Erythritol on the Antioxidant Capacity and Antidiabetic Properties of Shortbread Cookies. Pol. J. Food Nutr. Sci..

[5] Szpicer A., Onopiuk A., Wojtasik-Kalinowska I., Półtorak A. (2021). Red grape skin extract and oat β-glucan in shortbread cookies: Technological and nutritional evaluation. Eur. Food Res. Technol..

[6] Goubgou M., Songré-Ouattara L.T., Bationo F., Lingani-Sawadogo H., Traoré Y., Savadogo A. (2021). Biscuits: A systematic review and meta-analysis of improving the nutritional quality and health benefits. Food Prod. Process. Nutr..

[7] Fatima S., Altemimi A.B., Ali K., Inam-Ur-Raheem M., Mukhtar F., Yıkmış S., Abdi G., Aadil R.M. (2025). A comprehensive review on effect of chia seed, flaxseed, sesame seed and their derivatives on quality of bakery products. Appl. Food Res..

[8] Shatat N., Khalil M., El-Gammal R. (2023). Effect of adding veggies powdered on physico-chemical and sensory properties of cookies. Food Technol. Res. J..

[9] Kozlowska M., Zbikowska A., Marciniak-Lukasiak K., Kowalska M. (2019). Herbal extracts incorporated into shortbread cookies: Impact on color and fat quality of the cookies. Biomolecules.

[10] Desmiaty Y., Xavier F., Sandhiutami N.M.D., Noviani Y., Alatas F., Agustin R. (2025). Unlocking the potential of *Citrus aurantiifolia* bioactive compounds, functional benefits, and food applications: A comprehensive review. Food Biosci..

[11] Sharma R., Verma S., Rana S., Rana A. (2018). Rapid screening and quantification of major organic acids in citrus fruits and their bioactivity studies. J. Food Sci. Technol..

[12] Lin L.-Y., Chuang C.-H., Chen H.-C., Yang K.-M. (2019). Lime (*Citrus aurantifolia* (Christm.) Swingle) essential oils: Volatile compounds, antioxidant capacity, and hypolipidemic effect. Foods.

[13] Kadapatti L., Gorabal K., Mangesh, Kirankumar K.C., Mushrif S.K. (2025). Standardization of protocol for preparation of dehydrated whole lime fruits. Plant Arch..

[14] Hamadamin S.I. (2022). Effect of the dried limes (*Citrus aurantifolia*) on the caffeine kinetic extraction in black tea leaves (kinetic and thermodynamic study). Bull. Chem. Soc. Ethiop..

[15] Yadav A.R., Chauhan A.S., Rekha M.N., Rao L.J.M., Ramteke R.S. (2004). Flavour quality of dehydrated lime [*Citrus aurantifolia* (Christm.) Swingle]. Food Chem..

[16] Syed S.A., Bari A., Kaimkhani Z.A., Ali E.A. (2023). Inductively coupled plasma mass spectrometry assessment of essential and toxic trace elements in traditional spices consumed by the population of the Middle Eastern region in their recipes. Open Chem..

[17] Di Stasi M., Kaboudari A., Mancini S., Vacchina V., De Leo M., Braca A., Fratini F. (2025). Effect on food-related pathogens and chemical fingerprint of regional recipes of Baharat, a traditional Middle Eastern spice blend. Food Biosci..

[18] Altemimi A., Ali H.I., Al-Hilphy A.R.S., Lightfoot D.A., Watson D.G. (2018). Electric field applications on dried key lime juice quality with regression modeling. J. Food Process. Preserv..

[19] Aboud S.A., Altemimi A.B., Al-Hilphy A.R.S., Watson D.G. (2020). Effect of batch infrared extraction pasteurizer (BIREP)-based processing on the quality preservation of dried lime juice. J. Food Process. Preserv..

[20] Sasanka I., Wijewardane N.A., Wijesinghe W.A.J.P., Jeewanthi W., Priyadarshana I.B. (2025). Development of banana flour incorporated biscuit and evaluation of its physicochemical properties. Adv. Technol..

[21] Agócs A., Nagy V., Szabó Z., Márk L., Ohmacht R., Deli J. (2007). Comparative study on the carotenoid composition of the peel and the pulp of different citrus species. Innov. Food Sci. Emerg. Technol..

[22] Desmiaty Y., Sandhiutami N.M.D., Mulatsari E., Maziyah F.A., Rahmadhani K., Algifari H.O.Z., Jantuna F.A. (2024). Antioxidant and anti-inflammatory activity through inhibition of NF-κB and sEH of some citrus peel and phytoconstituent characteristics. Saudi Pharm. J..

[23] Izli N., Taskin O., Izli G. (2019). Drying of lime slices by microwave and combined microwave-convective methods. Ital. J. Food Sci..

[24] Ali A., Asgher Z., Cottrell J.J., Dunshea F.R. (2024). Screening and characterization of phenolic compounds from selected unripe fruits and their antioxidant potential. Molecules.

[25] Czech A., Malik A., Sosnowska B., Domaradzki P. (2021). Bioactive substances, heavy metals, and antioxidant activity in whole fruit, peel, and pulp of citrus fruits. Int. J. Food Sci..

[26] Ding S., Wang R., Zhang J., Li G., Zhang J., Ou S., Shan Y. (2017). Effect of drying temperature on the sugars, organic acids, limonoids, phenolics, and antioxidant capacities of lemon slices. Food Sci. Biotechnol..

[27] Xu G., Ye X., Chen J., Liu D. (2007). Effect of heat treatment on the phenolic compounds and antioxidant capacity of citrus peel extract. J. Agric. Food Chem..

[28] Papoutsis K., Pristijono P., Golding J.B., Stathopoulos C.E., Bowyer M.C., Scarlett C.J., Vuong Q.V. (2017). Effect of vacuum-drying, hot-air drying and freeze-drying on polyphenols and antioxidant capacity of lemon (*Citrus limon*) pomace aqueous extracts. Int. J. Food Sci. Technol..

[29] Phucharoenrak P., Muangnoi C., Trachootham D. (2023). Metabolomic analysis of phytochemical compounds from ethanolic extract of lime (*Citrus aurantifolia*) peel and its anti-cancer effects against human hepatocellular carcinoma cells. Molecules.

[30] Piccinelli A.L., García Mesa M., Armenteros D.M., Alfonso M.A., Arévalo A.C., Campone L., Rastrelli L. (2008). HPLC-PDA-MS and NMR characterization of C-glycosyl flavones in a hydroalcoholic extract of *Citrus aurantifolia* leaves with antiplatelet activity. J. Agric. Food Chem..

[31] Courts F.L., Williamson G. (2015). The occurrence, fate and biological activities of C-glycosyl flavonoids in the human diet. Crit. Rev. Food Sci. Nutr..

[32] Harborne J.B. (1965). Plant polyphenols—XIV. Characterization of flavonoid glycosides by acidic and enzymic hydrolyses. Phytochemistry.

[33] Hostetler G.L., Ralston R.A., Schwartz S.J. (2017). Flavones: Food sources, bioavailability, metabolism, and bioactivity. Adv. Nutr..

[34] Kim D.-S., Lim S.-B. (2020). Extraction of flavanones from immature *Citrus unshiu* pomace: Process optimization and antioxidant evaluation. Sci. Rep..

[35] Laganà V., Giuffrè A.M., De Bruno A., Poiana M. (2022). Formulation of biscuits fortified with a flour obtained from bergamot by-products (*Citrus bergamia*, Risso). Foods.

[36] Jongaroontaprangsee S., Tritrong W., Chokanaporn W., Methacanon P., Devahastin S., Chiewchan N. (2007). Effects of drying temperature and particle size on hydration properties of dietary fiber powder from lime and cabbage by-products. Int. J. Food Prop..

[37] Beuchat L.R. (1983). Influence of water activity on growth, metabolic activities and survival of yeasts and molds. J. Food Prot..

[38] Roudaut G., Dacremont C., Vallès Pàmies B., Colas B., Le Meste M. (2002). Crispness: A critical review on sensory and material science approaches. Trends Food Sci. Technol..

[39] Nawaz A., Li E., Khalifa I., Walayat N., Liu J., Nilofar, Muhammad Ahsan H., Irshad S., Barakat H., Lorenzo J.M. (2021). Effect of structurally different pectin on dough rheology, structure, pasting and water distribution properties of partially meat-based sugar snap cookies. Foods.

[40] Majzoobi M., Beparva P., Farahnaky A., Badii F. (2014). Effects of malic acid and citric acid on the functional properties of native and cross-linked wheat starches. Starch/Stärke.

[41] Bagagli M.P., Jazaeri S., Bock J.E., Seetharaman K., Sato H.H. (2014). Effect of transglutaminase, citrate buffer, and temperature on a soft wheat flour dough system. Cereal Chem..

[42] Miś A., Nawrocka A., Dziki D. (2017). Behaviour of dietary fibre supplements during bread dough development evaluated using novel farinograph curve analysis. Food Bioprocess Technol..

[43] Warren F.J., Gidley M.J., Flanagan B.M. (2016). Infrared spectroscopy as a tool to characterize starch ordered structure—A joint FTIR-ATR, NMR, XRD and DSC study. Carbohydr. Polym..

[44] Güven Ö., Şensoy İ. (2024). Effect of fibers on starch structural changes during hydrothermal treatment: Multiscale analyses, and evaluation of dilution effects on starch digestibility. J. Sci. Food Agric..

[45] Nawrocka A., Krekora M., Niewiadomski Z., Miś A. (2018). Characteristics of the chemical processes induced by celluloses in the model and gluten dough studied with application of FTIR spectroscopy. Food Hydrocoll..

[46] Sławińska A., Jabłońska-Ryś E., Gustaw W. (2024). Physico-chemical, sensory, and nutritional properties of shortbread cookies enriched with *Agaricus bisporus* and *Pleurotus ostreatus* powders. Appl. Sci..

[47] Kim E.H.-J., Corrigan V.K., Wilson A.J., Waters I.R., Hedderley D.I., Morgenstern M.P. (2012). Fundamental fracture properties associated with sensory hardness of brittle solid foods. J. Texture Stud..

[48] Arimi J.M., Duggan E., O’Sullivan M., Lyng J.G., O’Riordan E.D. (2010). Development of an acoustic measurement system for analyzing crispiness during mechanical and sensory testing. J. Texture Stud..

[49] Zarzycki P., Wirkijowska A., Teterycz D., Łysakowska P. (2024). Innovations in wheat bread: Using food industry by-products for better quality and nutrition. Appl. Sci..

[50] Lim E.-J. (2023). Study of the quality characteristics of cookies made with the addition of *Citrus unshiu* Markovich peel powder. J. East Asian Soc. Diet. Life.

[51] Cao W., Chen J., Li L., Ren G., Duan X., Zhou Q., Zhang M., Gao D., Zhang S., Liu X. (2022). Cookies fortified with *Lonicera japonica* Thunb. extracts: Impact on phenolic acid content, antioxidant activity and physical properties. Molecules.

[52] Schaich K.M., Tian X., Xie J. (2015). Hurdles and pitfalls in measuring antioxidant efficacy: A critical evaluation of ABTS, DPPH, and ORAC assays. J. Funct. Foods.

[53] Abdel-Aal E.-S.M., Rabalski I. (2022). Changes in phenolic acids and antioxidant properties during baking of bread and muffin made from blends of hairless canary seed, wheat, and corn. Antioxidants.

[54] Morales F.J., Martin S., Açar Ö.Ç., Arribas-Lorenzo G., Gökmen V. (2009). Antioxidant activity of cookies and its relationship with heat-processing contaminants: A risk/benefit approach. Eur. Food Res. Technol..

[55] Imran M., Butt M.S., Iqbal M.J., Gilani S.A., Basharat S., Saeed F., Suleria H.A.R. (2016). Antioxidant potential, physico-chemical, and sensory attributes of cookies supplemented with mosambi peel extract. Int. J. Fruit Sci..

[56] Kausar T., Saeed E., Hussain A., Firdous N., Bibi B., Kabir K., Ul An Q., Ali M.Q., Najam A., Ahmed A. (2024). Development and quality evaluation of cookies enriched with various levels of grapefruit pomace powder. Discov. Food.

[57] Al-Saab A.H., Gadallah M.G.E. (2021). Phytochemicals, antioxidant activity and quality properties of fibre enriched cookies incorporated with orange peel powder. Food Res..

[58] Shurvell H.F., Chalmers J.M., Griffiths P.R. (2006). Spectra–structure correlations in the mid- and far-infrared. Handbook of Vibrational Spectroscopy.

[59] Gómez A., Ferrero C., Calvelo A., Añón M.C., Puppo M.C. (2011). Effect of mixing time on structural and rheological properties of wheat flour dough for breadmaking. Int. J. Food Prop..

[60] Folorunso A.A., Soyebo K.O., Bakare K.O., Oladeji D. (2021). Quality improvement of biscuits using citrus peels and date palm fruits. Niger. J. Nutr. Sci..

[61] Krawęcka A., Sobota A., Pankiewicz U., Zielińska E., Zarzycki P. (2021). Stinging nettle (*Urtica dioica* L.) as a functional component in durum wheat pasta production: Impact on chemical composition, in vitro glycemic index, and quality properties. Molecules.

[62] Pérez-Jiménez J., Saura-Calixto F. (2005). Literature data may underestimate the actual antioxidant capacity of cereals. J. Agric. Food Chem..

[63] Re R., Pellegrini N., Proteggente A., Pannala A., Yang M., Rice-Evans C. (1999). Antioxidant activity applying an improved ABTS radical cation decolorization assay. Free Radic. Biol. Med..

[64] Shimamura T., Sumikura Y., Yamazaki T., Tada A., Kashiwagi T., Ishikawa H., Matsui T., Sugimoto N., Akiyama H., Ukeda H. (2014). Applicability of the DPPH assay for evaluating the antioxidant capacity of food additives—Inter-laboratory evaluation study. Anal. Sci..

[65] Krajewska A., Dziki D., Yilmaz M.A., Özdemir F.A. (2024). Broccoli pomace: Effect of drying methods and temperature on the grinding process and physicochemical properties. Int. Agrophys..

[66] Łubek-Nguyen A., Boguszewska-Czubara A., Nowacka-Jechalke N., Wójcik J., Olech M. (2026). Recovery of High-Value Compounds from Rose Root Biomass: Solvent Influence on Extraction Efficiency, Bioactivity, and Safety. Food Bioprod. Process..

[67] Dadalı C., Özcan Y., Ensari İ.C. (2025). Multifactorial optimization of gluten-free cookie with artichoke bracts as rice flour substitute and transglutaminase. Food Sci. Nutr..

[68] Krajewska A., Dziki D. (2025). Utilization of pear pomace as a functional additive in biscuit production: Physicochemical and sensory evaluation. Int. Agrophys..

[69] Kłosok K., Welc-Stanowska R., Nawrocka A. (2024). Effects of phenolic acid molecular structure on the structural properties of gliadins and glutenins. Int. Agrophys..

[70] Nawrocka A., Zarzycki P., Kłosok K., Welc R., Wirkijowska A., Teterycz D. (2023). Effect of dietary fibre waste originating from food production on the gluten structure in common wheat dough. Int. Agrophys..

[71] Nawrocka A., Szymańska-Chargot M., Miś A., Ptaszyńska A.A., Kowalski R., Waśko P., Gruszecki W.I. (2015). Influence of dietary fibre on gluten proteins structure—A study on model flour with application of FT-Raman spectroscopy. J. Raman Spectrosc..

[72] Stani C., Vaccari L., Mitri E., Birarda G. (2020). FTIR investigation of the secondary structure of type I collagen: New insight into the amide III band. Spectrochim. Acta A Mol. Biomol. Spectrosc..

