# Supplementary material for: Characterization of Black Lime (Citrus × aurantiifolia) Powder and Its Effects on the Physicochemical, Antioxidant, and Sensory Properties of Shortbread Cookies
Source: Molecules. 2026 Sep 15;31(18):3271. doi: 10.3390/molecules31183271 (PMC13609179; doi:10.3390/molecules31183271)
Supplement: Supplementary file 1 [file molecules-31-03271-s001.zip › molecules-4546649-supplementary.pdf]

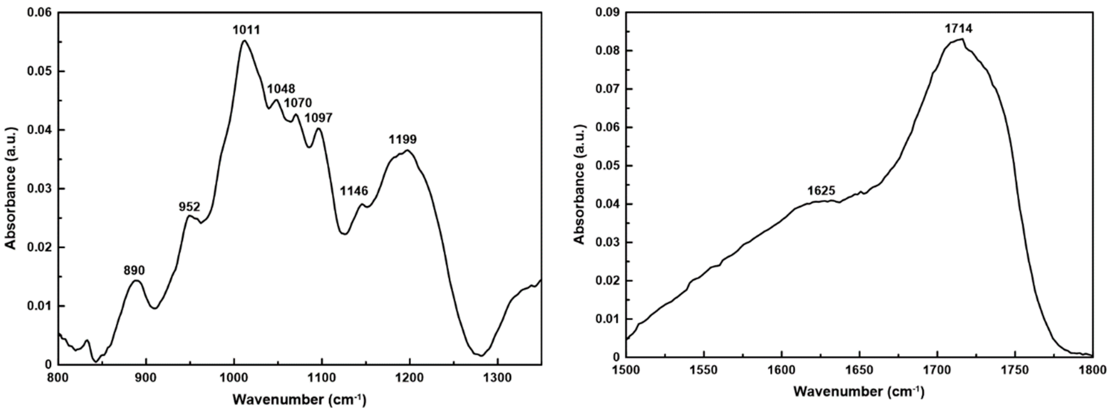

Figure S1. FTIR spectra of black lime powder in two spectral ranges: 800 – 1350 cm^{-1} And 1500 – 1800 cm^{-1}.

Table S1. Descriptive statistics of sensory evaluation of control and black lime powder-substituted samples (n = 46).

Sample	Parameter	Mean	Median	Minimum	Maximum	Lower	Upper	Range	Quartile	SD
**Control**	Appearance	7.50	7	6	9	7	8	3	1	0.81
	Smell	8.20	8	7	9	8	9	2	1	0.75
	Taste	7.80	8	6	9	7	8	3	1	0.78
	Color	8.00	8	6	9	8	8	3	0	0.70
	Texture	8.00	8	6	9	7	9	3	2	0.79
	Overall acceptability	8.30	8.5	6	9	8	9	3	1	0.81
**3BLP**	Appearance	7.39	8	5	9	7	8	4	1	1.00
	Smell	8.39	8	6	9	8	9	3	1	0.68
	Taste	8.00	8	6	9	8	9	3	1	0.76
	Color	7.50	8	6	9	7	8	3	1	0.81
	Texture	7.70	8	6	9	7	8	3	1	0.89
	Overall acceptability	8.11	8	7	9	8	9	2	1	0.74
**6BLP**	Appearance	7.20	7	5	9	7	8	4	1	1.02
	Smell	8.50	9	7	9	8	9	2	1	0.66
	Taste	8.50	9	7	9	8	9	2	1	0.69
	Color	7.61	8	5	9	7	8	4	1	0.88
	Texture	7.89	8	6	9	7	8	3	1	0.82
	Overall acceptability	8.30	8	6	9	8	9	3	1	0.73

Sample	Parameter	Mean	Median	Minimum	Maximum	Lower	Upper	Range	Quartile	SD
**9BLP**	Appearance	7.11	7	6	9	6	8	3	2	0.97
	Smell	8.70	9	7	9	8	9	2	1	0.55
	Taste	8.39	9	6	9	8	9	3	1	0.83
	Color	7.39	7	5	9	7	8	4	1	1.11
	Texture	8.20	8	6	9	8	9	3	1	0.81
	Overall acceptability	8.00	8	7	9	8	8	2	0	0.70
**12BLP**	Appearance	6.80	7	5	9	6	7	4	1	1.09
	Smell	8.20	8	6	9	8	9	3	1	0.75
	Taste	7.80	8	5	9	7	9	4	2	1.05
	Color	7.20	7	6	9	7	8	3	1	0.86
	Texture	7.89	8	6	9	7	9	3	2	0.90
	Overall acceptability	7.80	8	6	9	7	9	3	2	0.96
**15BLP**	Appearance	6.89	7	5	8	6	7	3	1	0.80
	Smell	7.89	8	6	9	7	8	3	1	0.85
	Taste	7.20	7	5	9	7	8	4	1	1.02
	Color	7.39	7	6	9	7	8	3	1	0.86
	Texture	7.50	7	6	9	7	8	3	1	0.84
	Overall acceptability	7.30	7	6	9	7	8	3	1	0.89

Control – sample without black lime powder addition; 3BLP–15BLP – samples with 3–15% replacement of wheat flour by black lime powder.

Table S2. Statistical groupings for sensory evaluation of control and black lime-enriched samples.

Sample	Appearance	Aroma	Taste	Color	Texture	Overall acceptability
**Control**	a	b	b	a	a	a
**3BLP**	a	a	a	a	a	a
**6BLP**	a	a	a	a	a	a
**9BLP**	a	a	a	b	a	a
**12BLP**	b	b	b	b	a	a
**15BLP**	b	b	c	b	b	b

Control – sample without black lime addition; BLP – black lime powder; 3BLP–15BLP – samples with 3–15% replacement of wheat flour by black lime powder. Different letters within the same column indicate significant differences between samples at $p < 0.05$.

Table S3. Formulation of control and BLP-enriched shortbread cookies (% w/w of total dough).

Ingredient	The Level of Wheat Flour Replacement (%)					
	Control	3BLP	6BLP	9BLP	12BLP	15BLP
Wheat flour	45	43.65	42.3	40.95	39.6	38.25
Butter	30	30	30	30	30	30
Sugar	13	13	13	13	13	13
Egg	12	12	12	12	12	12
BLP	0	1.35	2.7	4.05	5.4	6.75

Control – sample without black lime powder addition; 3BLP–15BLP – samples with 3–15% replacement of wheat flour by black lime powder.

Table S4. Analytical parameters used for quantitative LC-MS/MS analysis of polyphenols.

Compound	LOD [ng/mL]	LOQ [ng/mL]	R^2	Linearity range [ng/mL]
Gallic acid	600	1000	0.9992	1200-10000
5-Caffeoylquinic acid	80	160	0.9992	180-3600
Protocatechuic acid	150	300	0.9998	600-6000
4-Hydroxybenzoic acid	30	70	0.9995	70-1400
Gentisic acid	80	180	0.9993	200-2200
Vanillic acid	100	250	0.9997	330-33000
Syringic acid	170	390	0.9993	400-8000
3-Hydroxybenzoic acid	40	70	0.9994	100-2000
4-Hydroxycinnamic acid (*p*-coumaric acid)	19	40	0.9996	80-1500
Sinapic acid	18	40	0.9999	80-1400
Ferulic acid	17	35	0.9994	70-1400
Salicylic acid	250	500	0.9991	500-5000
Eriodyctiol	7	13	0.9990	30-6000
Luteolin	8	16	0.9990	20-400
Quercetin	40	70	0.9992	70-1400
Apigenin	15	30	0.9989	30-6000
Isorhamnetin	120	224	0.9991	250-4000
Eriocitrin (Eriodictyol-7-*O*-rutinoside)	250	500	0.9995	1000-12000
Apigenin – 6-*C*-glucoside (Isovitexin)	80	150	0.9991	500-5000
Apigenin – 8-*C*-glucoside (Vitexin)	82	160	0.9992	250-2500
Naringenin 7-*O*-glucoside	100	200	0.9996	500-10000

Compound	LOD [ng/mL]	LOQ [ng/mL]	R^2	Linearity range [ng/mL]
Quercitrin (Quercetin 3-*O*-rhamnoside)	60	130	0.9991	250-5000
Narcissoside (Isorhamnetin 3-*O*-rutinoside)	50	100	0.9990	200-4000
Luteoloside (Luteolin-7-*O*-glucoside)	100	190	0.9989	250-5000
Eriodictyol-7-glucopyranoside	65	120	0.9994	500-5000
Naringenin-7-*O*-rutinoside (Narirutin)	90	160	0.9994	500-1500
Isorhamnetin 3-O-glucoside	100	180	0.9995	350-3500
Apigenin 7-*O*-glucoside	110	200	0.9997	250-1670

Table S5. Thickness and diameter of shortbread cookies.

Sample	Thickness [mm]	Diameter [mm]
Control	8.18 ± 0.23[ab]	44.82 ± 0.61[a]
3BLP	8.37 ± 0.25[a]	44.47 ± 0.51[a]
6BLP	8.27 ± 0.25[a]	44.40 ± 0.81[a]
9BLP	8.08 ± 0.27[ab]	44.31 ± 0.40[a]
12BLP	7.96 ± 0.18[b]	44.51 ± 0.43[a]
15BLP	7.90 ± 0.20[b]	44.46 ± 0.78[a]

BLP – black lime powder; 3BLP–15BLP – samples with 3–15% replacement of wheat flour by black lime powder.
